# Sociodemographic differences in the cultural significance of edible and toxic mushrooms among Tsotsil towns in the Highlands of Chiapas, Mexico

**DOI:** 10.1186/s13002-018-0232-9

**Published:** 2018-05-03

**Authors:** Felipe Ruan-Soto

**Affiliations:** Centro de Investigaciones Multidisciplinarias sobre Chiapas y la Frontera Sur, Universidad Nacional Autónoma de México, San Cristóbal de Las Casas, México

**Keywords:** Ethnomycology, Ethnobiology, Local mycological knowledge, Mushroom intoxications, Mycetism

## Abstract

**Background:**

Mushrooms are important forest resources, mostly as food, despite the serious health threat posed by toxic species. In the Highlands of Chiapas, numerous wild mushroom intoxications have been registered. While Chiapas has been vastly studied from an ethnomycological perspective, no certainty exists as to how nomenclature systems differentiate edible and toxic species, which species are most culturally significant, and whether sociodemographic factors relate to how well-known they are in the Highlands of Chiapas. This paper evaluates which are the most culturally significant edible and toxic wild mushroom species in seven Tsotsil communities from this region and whether differences exist in their knowledge relating to different sociodemographic subsets (gender, schooling, and occupation). The hypothesis that there is a difference in the number of species that people mention, as well as the number of times each ethno-taxon is mentioned, between people from different social groups was tested.

**Methods:**

With consent, 133 Tsotsil people from seven communities were interviewed. Interviews focused on local systematics and free listings of edible and toxic mushrooms. Qualitative and quantitative analyses were performed, including multivariate methods and non-parametric statistics.

**Results:**

Twenty-five edible and 15 toxic taxa were mentioned. Some directly correspond to Linneanean species, while others are subdifferentiated or supradifferentiated. Only 62% of the interviewees named toxic mushrooms. The most frequently mentioned edible taxa were *Amanita hayalyuy* and *A. jacksoniii*, *Agaricus* spp., and *Armillaria mellea*. The most frequently mentioned toxic species were *Amanita muscaria*, *Suillellus luridus*, and *Russula emetica*. Significant differences in the number of mentioned edible ethnotaxa were found only among different occupations and schooling. The models including schooling interacting with either gender or occupation are better supported. Significant differences in the number of times toxic ethnotaxa are mentioned were found only between men and women.

**Conclusions:**

The Tsotsil region of the Highlands of Chiapas is where the most average mushroom species are recognized state-wide. Schooling and occupation seem most determinant for people to know more or less species of mushrooms, while gender appears irrelevant. People with no studies and field-related occupations name more species. Identification criteria to distinguish edible from toxic species seem to rest not on detailed recognition of the second set but precise knowledge of the first.

## Background

Mushrooms are without a doubt a greatly relevant non-timber forest resource for human communities around the world [1–3]. These organisms have been used throughout history as medicine, amulets, fodder, combustible, ritualistic materials, and mostly, as food [4]. Local knowledge has been developed regarding wild edible mushrooms, their ecology, phenology, and morphology; this has permitted their use [5–7]. In Mexico, this is the case to such an extent that a little under 400 species have been registered as edible [8].

However, not all edible species are equally significant; preference of certain species or groups of species is always present [9]. Hunn [10] defined the cultural significance of a taxon as the value or role that organism plays within a given culture. To quantitatively evaluate the said significance, many indexes and indicators have been designed for plants, animals, and of course, mushrooms [11–17]. For the latter, different studies have recorded the most significant edible species for inhabitants of diverse communities and the relation between the said importance and sociodemographic factors such as gender, occupation, and age [14, 15].

However, forest contain not only edible species, but also toxic mushroom species that can be morphologically similar to edible species and so cause confusion among collectors [18]. Their occasional ingestion causes health problems from gastrointestinal discomfort and serious diarrheic spells to death [19]. The intoxications provoked by the accidental consumption of wild toxic species are known as mycetisms [20]. Because of this, the proper recognition of mushroom species and the differentiation of toxic and edible species is of vital importance for the safe use of wild mushrooms.

To different authors, the very act of naming a species reflects the fact that they have been used somehow and are of interest to the human group in question [10, 21]. Since only a part of the natural discontinuities is recognized in ethnobiological classification, organisms of cultural significance would be known only in very general terms [10]. The cultural significance of wild edible mushrooms is quite clear; several ethnomycological studies have focused in local taxonomy and systematics as well as significance itself [22–24]. Contrastingly, the local nomenclature, systematics, and cultural significance of toxic mushrooms have seldom been the focus of research [18]. Nonetheless, it is not unlikely that these species are significant since the prevention of intoxications depends on their recognition.

In the Highlands of Chiapas, southeast Mexico, particularly among the Tsotsil Maya, several intoxications from wild toxic mushroom consumption has been registered over the last 10 years [25, 26]. Specifically, between 2005 and 2013, there have been 35 intoxication events in this region, 31 were fatal [26]. In response, the health authorities in Chiapas have resorted to prohibition without considering biocultural aspects. While Chiapas is one of the most studied states in Mexico from an ethnomycological point of view [27], there is no certainty to date as to which wild edible and toxic species are the most culturally significant in the Highlands of Chiapas. Furthermore, there is little knowledge about their traditional nomenclature systems and whether sociodemographic factors play a part in the degree to which people know these species.

This paper evaluates which are the most culturally significant edible and toxic wild mushroom species in seven Tsotsil communities from this region and whether differences exist in their knowledge relating to different sociodemographic subsets (gender, schooling, and occupation). The hypothesis that there is a difference in the number of species that people mention, as well as the number of times each ethno-taxon is mentioned, between people from different social groups was tested.

## Methods

### Study site

The geographic, economic, and sociocultural area known as “los Altos de Chiapas” (the Highlands of Chiapas) comprises 17 municipalities spanning 3770 km^2^ [28]. It is located in the northwestern portion of the Mexican southern frontier state, Chiapas. As the northernmost limit of the Sierra Madre de Chiapas mountain range, it is made up of elevations and valleys with altitudes throughout the region varying from 1200 to 2700 m a.s.l. [29]. Climates in this area include Cw2, Cm, and C(A)w. Annual precipitation ranges from 1300 to 2200 mm [30].

Vegetation in this region is dominated by pine (*Pinus* spp.), oak (*Quercus* spp.), and liquidambar (*Liquidambar* spp.) species [31]. While literature reports the presence of communities such as temperate forests with oak dominance, and mist forests in the region, Woodland areas have undergone a dramatic transformation over the last few decades in favor of non-forest, cultivated, and pine-dominated areas [32].

This region has a population of 645,099 inhabitants [28]. The dominant ethnic groups are Tsotsil and Tseltal who make up 68% of the total population. The main economic activities in the zone are agriculture, tourism, and commerce [30].

**Fig. 1:**
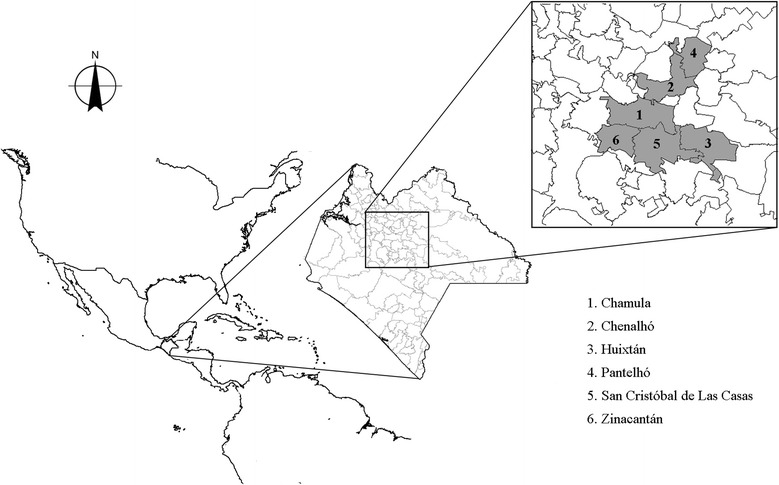
Location of the study sites in the Highlands of Chiapas, Mexico

Specifically, fieldwork was carried out in the municipalities: Chamula (central west and east) (16° 47′ N 92° 41′ W,), Chenalho (16° 53′ N 92° 38′ W), Huixtan (16° 46′ N 92° 27′ W), Pantelho (17° 0′ N 92° 29′ W), San Cristobal de Las Casas (16° 44′ N 92° 38′ W), and Zinacantan (16° 45′ N 92° 43′ W) (Fig. 1). The mean altitude of these municipalities spans from 1597 m a.s.l. (Chenalho) to 2286 m a.s.l. (Chamula) [28]. In these sites, with the exception of San Cristobal de Las Casas, population is predominantly Tsotsil.

### Data collection and analysis

Prior to field work, previous, free, and informed consent was obtained by political and traditional authorities in the seven study sites in order to apply interviews among the people willing to participate, to collect mushrooms, and to publish results and images from this research. All work was carried out in accordance to the principles of the ethical code for the Latin American Society of Ethnobiology [33].

From May to August 2017 structured and semi-structured interviews [34] were carried out with 133 haphazardly chosen people. All of the interviewed population was over 20 years old and spoke Tsotsil as their first language; they were from the communities Chamula (west-center) (21), Chamula (east) (19), Chenalho (15), Huixtan (23, Pantelho (15), San Cristobal de Las Casas (18), and Zinacantan (22). Interviews were carried out in Spanish and assisted by a translator; additionally, they were recorded digitally for a later literal translation. Semi-structured interviews dealt mainly with local taxonomy and classification, as well as ethnoecological knowledge and use of mushrooms. Structured interviews contained (a) sociodemographic information (gender, school level, occupation, age, community, and municipality of residence); (b) two free listings in which the interviewed were asked to name all the edible and toxic species they knew; (c) taxonomic corroboration of local names and biological species. This last exercise was accomplished with the aid of a photographic catalog with images of 30 of the most frequently mentioned ethnotaxa from ethnomycological studies in the Highlands of Chiapas and 17 reported toxic species for the same region [35–45]. This catalog was designed following suggestions from Thomas et al. [46] regarding image proportion, size, and definition.

Semistructured interviews were analyzed by constant comparison of analysis categories as is proposed by Sandoval [47]. The frequency of mention was used as an indicator of cultural significance both for edible and toxic species. Thus, the most mentioned ethnotaxon in interviews was deemed the most significant [15, 16, 48]. In order to determine significant differences between gender, occupation, and school level, the interviewed population was divided as follows: (a) by gender: women and men; (b) by occupation, the population was classified as proposed by Saynes-Vasquez et al. [49]: those with occupations linked to the field, that is primary productive activities (mainly peasants); secondly, those with occupations not linked to the field, namely secondary activities related to resource transformation and processing; and finally, those involved in tertiary activities, namely services (in this group, laborers, merchants, chauffeurs, and people with a career are included); (c) by schooling people were classified as no schooling (those lacking any form of formal education) and people with schooling (those who have at least a basic formal education).

Mann-Whitney tests were performed using these groups to determine the presence of differences in the number of mentioned species, both edible and toxic. The number of times each ethnotaxon was mentioned were compared through *χ*^2^ tests. In order to evaluate the interactions between these conditions, several models using the beta probability-density function were built [50]. Each model included one or more beta distributions that described the probability density of observing an individual with a given number of known edible/toxic mushrooms in a population having a determined sociocultural attribute. In total, five models were constructed by fitting through maximum likelihood a beta distribution to different subsets of the number of known edible/toxic mushrooms values sampled: (a) Null model: The probability of sampling a person with any given number of known edible/toxic mushrooms is independent of the sociocultural variables. (b) Two-factor models: including a combination of two sociodemographic features (gender-occupation, gender-education and occupation-education). (c) Three-factor model: including a combination of three features (gender-occupation-education). The models were then compared with the Akaike information criterion (AIC). This procedure allows for a hierarchical organization of the models that formally indicates the evidence supporting each one, thus permitting a selection of the best competing model. If any model has an AIC value two units lower than another, it is concluded that the former is better supported by the data. If the difference in AIC values is smaller than two units, both models have similar support and it is impossible to select one over the other [51].

Finally, a distance matrix was constructed to calculate the mean taxonomical distance to explore differences between the study sites based on the relative frequency of mention of edible and toxic mushrooms. With these values, cluster analyses were performed using the UPGMA method and a principal component analysis (PCA) was carried out using NTSYS ver. 2.11 for PC [52] to explore variation patterns in the free listing responses.

## Results

Considering all seven Tsotsil study sites in the Highlands of Chiapas, people mentioned 25 edible taxa in free listing exercises (Table 1). With the aid of a photographic catalog, the interviewed population recognized 28 taxa (Table 2).

**Table 1:** Frequencies of mention of the edible taxa registered in the study sites

Taxa	No. Men.	rel FM	rel FM. M	rel FM. W	Dif. M-W	rel FM. F	rel FM. NF	Dif. F-NF	rel FM. E	rel FM. NE	Dif. E-NE
*Agaricus* spp.	95	71.43	66.13	76.06	−9.93	78.95	65.79	13.16	60.26	76.36	−16.11
*Amanita hayalyuy D. Arora & G.H. Shepard y Amanita jacksonii Pomerl.*	114	85.71	85.48	85.92	−0.43	85.96	85.53	0.44	74.36	90.91	−16.55
*Amanita vaginata* (Bull.) Lam.	13	9.77	8.06	11.27	−3.20	15.79	5.26	10.53	5.13	14.55	−9.42
*Armillaria melleak* (Vahl) P. Kumm.	72	54.14	46.77	60.56	−13.79	61.40	48.68	12.72	38.46	67.27	−28.81
*Auricularia* spp.	15	11.28	19.35	4.23	15.13	15.79	7.89	7.89	15.38	5.45	9.93
*Boletus* spp. y *Suillus* spp.	65	48.87	53.23	45.07	8.16	66.67	35.53	31.14	42.31	52.73	−10.42
*Calvatia* spp.	15	11.28	17.74	5.63	12.11	14.04	9.21	4.82	11.54	9.09	2.45
*Cantharellus cibarius s.l.*	32	24.06	25.81	22.54	3.27	19.30	27.63	−8.33	19.23	30.91	−11.68
*Clitocybe infundibuliformis* (Schaeff.) Quél.	5	3.76	3.23	4.23	−1.00	0.00	6.58	−6.58	1.28	7.27	−5.99
*Daldinia* spp.	11	8.27	3.23	12.68	−9.45	7.02	9.21	−2.19	5.13	10.91	−5.78
*Favolus tenuiculus* P. Beauv.	5	3.76	8.06	0.00	8.06	7.02	1.32	5.70	6.41	0.00	6.41
*Hydnum* spp.	11	8.27	8.06	8.45	−0.39	12.28	5.26	7.02	8.97	7.27	1.70
*Hypomyces lactifluorum* (Schwein.) Tul. & C. Tul.	38	28.57	29.03	28.17	0.86	40.35	19.74	20.61	19.23	38.18	−18.95
*Laccaria* spp.	32	24.06	17.74	29.58	−11.84	31.58	18.42	13.16	15.38	32.73	−17.34
*Lactarius deliciosus* s.l.	40	30.08	25.81	33.80	−8.00	38.60	23.68	14.91	24.36	30.91	−6.55
*Lactarius indigo* (Schwein.) Fr.	37	27.82	29.03	26.76	2.27	40.35	18.42	21.93	30.77	21.82	8.95
*Lentinus* spp.	4	3.01	0.00	5.63	−5.63	7.02	0.00	7.02	1.28	5.45	−4.17
*Lepista* sp.	11	8.27	11.29	5.63	5.66	10.53	6.58	3.95	3.85	14.55	−10.70
*Macrolepiota procera* (Scop.) Singer	1	0.75	1.61	0.00	1.61	0.00	1.32	−1.32	1.28	0.00	1.28
*Neolentinus lepideus* (Fr.) Redhead & Ginns	51	38.35	37.10	39.44	−2.34	49.12	30.26	18.86	33.33	40.00	−6.67
*Pleurotus djamor* (Rumph. ex Fr.) Boedijn	71	53.38	53.23	53.52	−0.30	52.63	53.95	−1.32	46.15	58.18	−12.03
*Ramaria* spp.	68	51.13	51.61	50.70	0.91	52.63	50.00	2.63	32.05	67.27	−35.22
*Schizophyllum commune* Fr.	21	15.79	19.35	12.68	6.68	21.05	11.84	9.21	15.38	14.55	0.84
*Tremella* spp.	2	1.50	1.61	1.41	0.20	1.75	1.32	0.44	1.28	1.82	−0.54
*Turbinellus floccosus* (Schwein.) Earle ex Giachini & Castellano	1	0.75	1.61	0.00	1.61	1.75	0.00	1.75	1.28	0.00	1.28

*No. Men.* number of mentions, *FM rel* relative frequency of mention, *rel FM. M* relative frequency of mention men, *rel FM. W* relative frequency of mention women, *rel FM. F* relative frequency of mention field-linked occupation, *rel FM. NF* relative frequency of mention non-field-linked occupation, *rel FM. E* relative frequency of mention formal education, *rel FM. NE* relative frequency of mention without formal education, *Dif.M-W* difference between men and women, *Dif. F-NF* difference between field-linked occupation and non-field-linked occupation, *Dif.E-NE* difference between formal education and without formal education

**Table 2:** Names given to edible mushrooms in the study site. The most common name for each ethnotaxon is in bold letters

Taxa	Central-west Chamula	East Chamula	Chenalho	Huixtan	Pantelho	San Cristobal de Las Casas	Zinacantan
*Agaricus* spp.	***Moni’***	*Moni’*	*Moni’,* Jonguillo	*Moni’, Konkilio*	*Moni’,* Jonguillo	*Moni’,* Jonguillo	*Moni’*
*Amanita hayalyuy* D. Arora & G.H. Shepard y *Amanita jacksonii* Pomerl.	***Yuy***/*Tsajal yuy*	*Yuy*/ *K’antsu*	*Yuy*	*Yuy, K’antsu*	*–*	*Yuy*	*Yuy*
*Armillaria mellea* (Vahl) P. Kumm.	***Chevev****, Checheval tulan/ Checheval chijilte’/ Checheval* San Andrés/ *Vixil chechev/ Mukil chechev*	*Chevev, Checheval tulan/ Chuchal chijilte’/ Vixil chechev/ Mukil chechev*	*–*	*Chechev*	*–*	*Chechev, Chechev* San Andrés	*Chechev, Chechev* San Andrés
*Boletus* spp.y *Suillus* spp.	***Sekub t’ul****,* Pancito	*Pan chuch/Sekub t’ul*	*Sekub t’ul,* Pancito	*P’ukus, P’ukuts t’ul, P’ukuts chuch*	*–*	*Sekub t’ul,* Semita	*Sekub t’ul*
*Cantharellus cibarius s.l.*	***Xmanayok***	*Xmanayok, K’anal chuch*	*Xmanayok*	*Xmanayok, K’an chay*	*–*	*Xmanayok*	*Xmanayok*
*Clitocybe infundibuliformis* (Schaeff.) Quél.	*Sakil chechev*	*Chikin vinajel*	*–*	*–*	*–*	*–*	*Sak balum,* ***Sak vinajel***
*Turbinellus floccosus* (Schwein.) Earle ex Giachini & Castellano	*Santa Roxa chuch*	*–*	*–*	***Chikin toro****,* Corneta	*–*	*–*	*–*
*Hydnum* spp.	***Yok wakax*** *, Yok sup, Yok sarut*	*Yok sup, Yok max, Yok vakax*	*Yok mis*	*Ch’ix manayok, Yok vakax*	*–*	*Yok wakax*	*Ch’ix manayok, Yok wakax*
*Helvella* spp.	*–*	*–*	*–*	*–*	*–*	*–*	*Chinam chi*
*Hypomyces lactifluorum* (Schwein.) Tul. & C. Tul.	***Chakat’ob***	*Chakat’ob*	*Chakat’ob*	*Tsajal ve’lil, Kamusa, Chikin chitom*	*–*	Chaquetón, Cresta de gallo	*Chakat’ob*
*Laccaria* spp.	***Kavixtoj*** *, Yax vinajel*	*Kavixtoj*	*–*	*Majarero, Kavixtoj*	*–*	*Kavixtoj*	*Kavixtoj*
*Lactarius deliciosus s.l.*	***K’anal manayok***	*K’anal chuch, K’anal manayok*	*–*	*K’anchay, K’anal chuch, K’anal manayok*	*–*	*K’anal manayok*	*K’anal manayok, Tsalum kelem*
*Lactarius indigo* (Schwein.) Fr.	***Yaxal manayok***	*Yaxal chuch*	*Yaxal manayok*	*Yaxal Chuch, Yaxal borran, Borran tuluk, K’anchay azul, Yaxal ve’lil, Yaxal manayok*	*–*	*Yaxal manayok*	*Yaxal chuch, Yaxal kelem*
*Morchella* spp.	***Mochilum wakax***	*Mochilum wakax*	*Kol kox*	*–*	*–*	*–*	*–*
*Neolentinus lepideus* (Fr.) Redhead & Ginns	***Taj chuch***	*Taj chuch*	*–*	*Taj chuch*	*Taxo*	*Taxo*	*Taj chuch*
*Ramaria* spp.	***Yisim chij***	*Yisim chij, Xulub chij*	*Yisim chij*	*Yisim chij, Xulub chij*	*–*	*Yisim chij*	*Yisim chij*
*Daldinia* spp.	*Yon ton tuluk, Chik te’, Chikin te’*	*Vuch tuluk*	*Vuch tuluk, Chik te’*	***T’ot*** *, Chikin te’*	*–*	*T’ot*	*T’ot*
*Calvatia* spp.	***Sat Pukuj***	*Sat pukuj*	*–*	*Chinam vakax*	*–*	*Sat pukuj*	*Yon ton tuluk, Vuch tuluk*
*Ustilago maydis* (DC.) Corda	***Tok*** *, Tokal ixim, Stokal chomtik,*	*Tok, Tokal ixim, Chuchal ixim*	*Tok*	*Xu’ Xu’ ixim*	*Stokal ixim,* Huitlacoche	*Stokal ixim,* Huitlacoche*,* Nanaguate	*Xu’ ixim*
*Auricularia* spp.	***Lolo pik’***	*Lolo pik’*	*Lolo pik’*	*K’o’ chikin, Korech*	*Lolo pik’*	*–*	*–*
*Pleurotus djamor* (Rumph. ex Fr.) Boedijn	***Sakitaj***	*Sakitaj*	*Chikin te’, sakitaj*	*Sakitaj*	*Chikin te’, Sakitaj*	*–*	*Sakitaj*
*Schizophyllum commune* Fr.	***Usum***	*Usum, Usum te’, Chuchal te’*	*Usum*	*Sulte’*	*Usum pik’*	*Usum*	*Usum, Kusum*
*Favolus tenuiculus* P. Beauv.	*–*	*–*	*Joch on pat*	***Sakil p’ukuts***	*–*	*–*	*–*
*Lentinus* spp.	*Tsutsuru*	***Nukul chikin***	*–*	*Nukul chuch, Uskun*	*–*	*–*	*–*
*Tremella* spp.	***Chikin ik’al***	*Chikin ik’al, Lolo pik’*	*–*		*–*	*–*	*Chikin ik’al*
*Amanita vaginata* (Bull.) Lam.	***Ik’al yuy***	*Ik’al yuy*	*–*	*Ik’al yuy*	*–*	*–*	*–*
*Macrolepiota procera* (Scop.) Singer	***Vixil moni’***	*–*	*–*	*–*	*–*	*–*	*–*
*Lepista* sp.	***Checheval mail***	*Chuchal mail*	*–*	*–*	*–*	*–*	*–*

Twelve edible ethnotaxa have a one-to-one correspondence to Linneaean species as defined by Berlin et al. [53]. Twelve ethnotaxa are underdifferentiated, since they include different species, in most cases, from a single genus. Lastly, *Armillaria mellea* (Vahl) P. Kumm. is over-differentiated, that is, it is locally recognized as different types of mushrooms (*chechev*) in the local perspective. Thus, it is named as varieties of *chechev: checheval tulan* and *checheval chiji’te*, according to the tree in which they grow (*Quercus* spp. and *Sambucus Mexicana* C. Presl. respectively), *checheval San Andres* because of the season in which it appears (around the day of Saint Andrew in November), or *vixil chechev* and *mukil chechev* according to its size (the first is bigger than the second).

In Table 2, the different names assigned to edible species in the studied Tsotsil municipalities can be viewed.

**Fig. 2:**
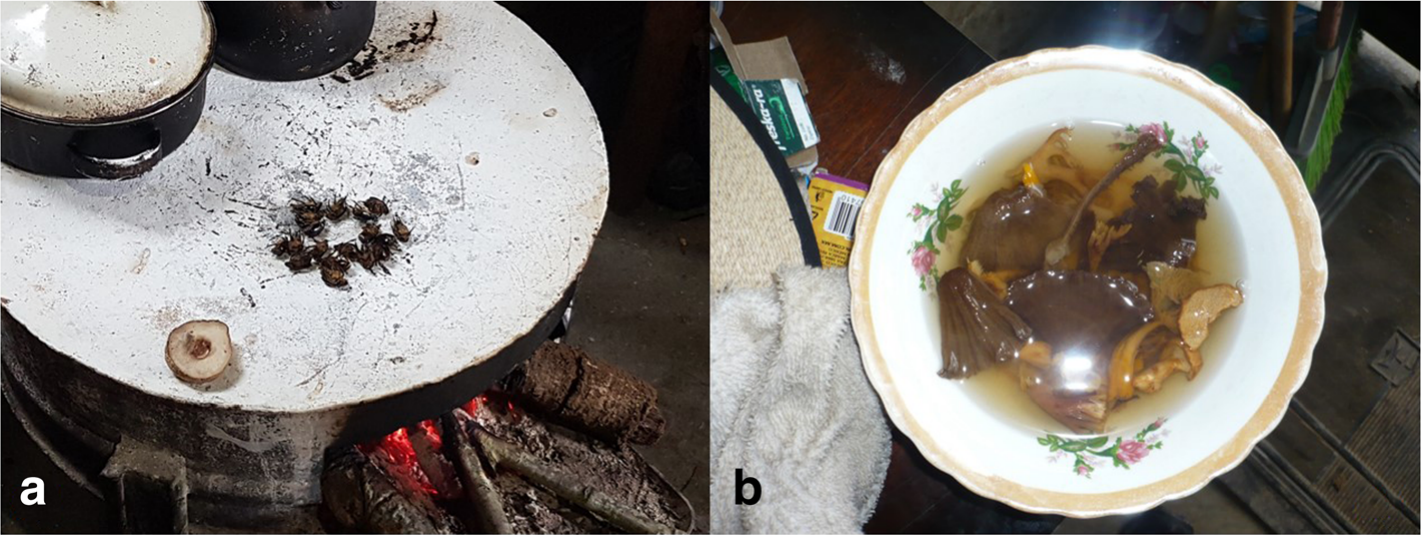
Preparation methods for edible mushrooms. **a**) Comal broiled *sekub t'ul* (*Boletus* sp.), **b**) Mixed mushroom broth

The most frequently mentioned edible taxa are *yuy* (*Amanita hayalyuy* D. Arora & G.H. Shepard and *A. jacksonii* Pomerl.), which was mentioned 114 times; *moni’* (*Agaricus* spp.), mentioned 95 times, *chechev* (*Armillaria mellea*), mentioned 72 times (Table 1). *Yuy* was mentioned by 85% of the interviewed population, in addition to being one of the most sold species in the region. In average, each interviewed person mentioned 6.24 edible species, with the lowest number of mentioned species at one and the largest at 17. Fifteen edible ethnotaxa are recognized by over 10% of the population and five of them are mentioned by over 50% of the people. In general, edible mushrooms are consumed separately in simple preparations such as broths or roasted on a *comal* (Fig. 2).

When we consider gender as a sociodemographic condition to look for differences in knowledge of edible species, the number of ethnotaxa mentioned by men and women is not significantly different when evaluated through a Mann-Whitney test (*U* = 0.7052, *P* > 0.05) (Table 3). However, men and women significantly differ in the number of times they mention each ethnotaxon (*χ*^2^ = 51.164, *P* = 0.0010) (Table 3). For example, *Auricularia* spp. is mentioned 15% more frequently by men than by women. In contrast, *Armillaria mellea* is mentioned 13% more frequently by women than by men (Table 1).

**Table 3:** Statistical tests to evaluate significant differences in the number of mentioned ethnotaxa and the number of times each ethnotaxon was mentioned in each sociodemographic group

Condition	Category	Variable	Median	Mann-Whitney	*χ* ^2^
Gender	Edible	Men	5.5	U = 0.7052 > 0.05	*χ*^2^ = 51.164, P = 0.0010
		Women	6		
	Toxic	Men	1	U = 0.1997 > 0.05	*χ*^2^ = 19.398, P = 0.1503
		Women	1		
Occupation	Edible	Field activities	7	*U = 0.0014 < 0.05*	*χ*^2^ = 51.682, P = 0.0009
		Non-field activities	5		
	Toxic	Field activities	1	U = 0.1754 > 0.05	*χ*^2^ = 30.385, P = 0.0068
		Non-field activities	1		
Schooling	Edible	Formal education	5	*U = 0.0162 < 0.05*	*χ*^2^ = 52.212, P = 0.0007
		No formal education	6		
	Toxic	Formal education	1	U = 0.0627 > 0.05	*χ*^2^ = 32.664, P = 0.0032
		No formal education	1		

Significant values appear in italics

Now, when occupation is compared, the number of edible ethnotaxa mentioned by people with occupations linked to the field is significantly greater than that mentioned by people with occupations not related to the field (*U* = 0.0014, *P* < 0.05) (Table 3). Furthermore, people working in the field and those who do not differ significantly in the number of times they mention each ethnotaxon (*χ*^2^ = 51.682, *P* = 0.0009) (Table 3). For example, *Boletus* spp. and *Suillus* spp. are mentioned 31% more frequently by people working in the field (Table 1).

When analyzing the school level of the interviewed population, the number of mentioned edible ethnotaxa is significantly less for people who have had formal studies than for those without formal education (*U* = 0.0162, *P* < 0.05) (Table 3). Furthermore, people with schooling and people without significantly differ in the number of times in which they mention each ethnotaxon (*χ*^2^ = 52.212, *P* = 0.0007). For example, *Ramaria* spp. is mentioned 35% more frequently by people without formal education than by people with (Table 1).

**Fig. 3:**
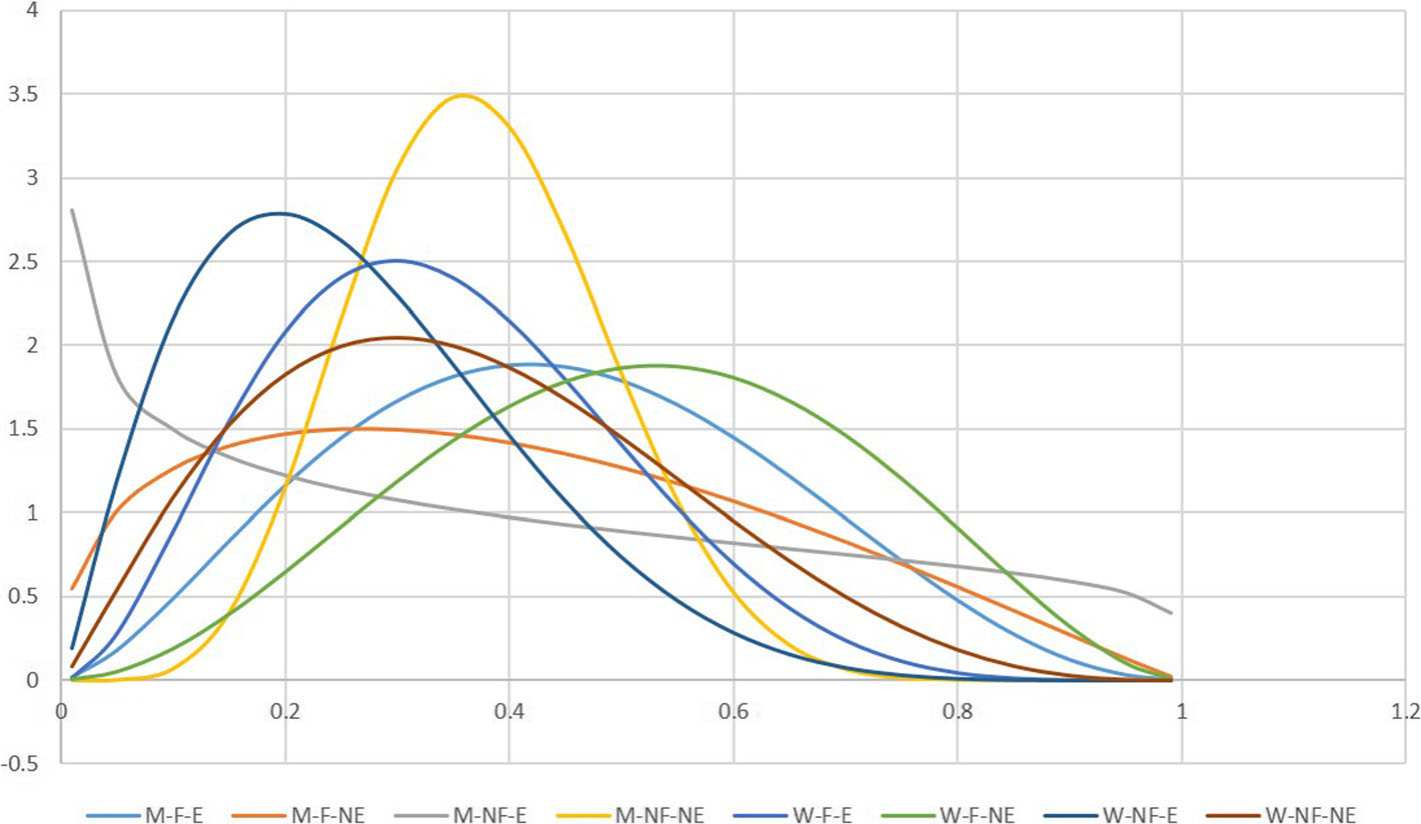
Probability density of the relative number of known edible mushrooms. Model including different sociodemographic features. M-F-E = Men-occupation linked to Field-with Education, M-F-NE = Men-occupation linked to Field-without Education, M-NF-E = Men-occupation Not linked to the Field-with Education, M-NF-NE = Men-occupation Not linked to the Field-without Education, W-F-E = Women-occupation linked to Field- with Education, W-F-NE = Women-occupation linked to Field-without Education, W-NF-E = Women-occupation Not linked to the Field-with Education, W-NF-NE = Women-occupation Not linked to the Field-without Education

Looking at different models to explore the interaction between sociodemographic conditions, those including education combined with either gender or occupation are better supported (Table 4). However, the best supported model is the one considering the interaction of all three features. Thus, there is a higher probability of finding unschooled field workers who recognize a high number of mushroom species regardless of their gender (Fig. 3).

**Table 4:** Models and AIC values for edible and toxic mushrooms

Models	AIC values edible mushrooms	AIC values toxic mushrooms
Null	−43.8584	−500.7521
MenField-MenNoField-WomenField-WomenNoField	−50.9813	− 499.6858
MenEducation-MenWithoutEducation-WomenEducation-WomenWithoutEducation	−53.3707	*− 502.0485*
FieldEducation-FieldWithoutEducation-NoFieldEducation-NoFieldWithoutEducation	−54.9691	*− 503.0055*
MenFieldEducation-MenFieldWithoutEducation-MenNoFieldEducation-MenNoFieldWithoutEducation- WomenFieldEducation-WomenFieldWithoutEducation-WomenNoFieldEducation-WomenNoFieldWithoutEducation-	*−61.7279*	− 480.5702

Significant values appear in italics

**Fig. 4:**
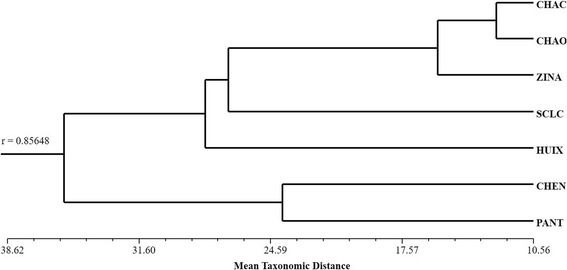
Cluster analysis of the four study sites using the Average Taxonomic Distance index based on the relative frequency of mention of edible mushrooms

**Fig. 5:**
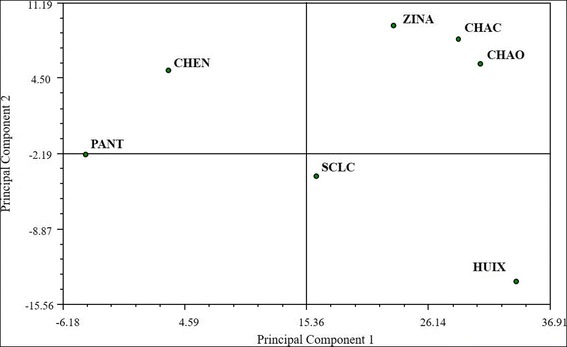
Principal component analysis for the studied communities based on the relative frequency of mention of edible mushrooms

The classification analysis based on the relative frequency of mention of edible mushrooms shows a variation pattern that may relate to the geographical space that these municipalities occupy (Fig. 4). One group includes east Chamula, center-west Chamula, and Zinacantan. Another group includes Chenalho and Pantelho. The PCA shows that the first principal component explains 40.20% of the variation, discriminating Chenalho and Pantelho from the rest of the communities (Fig. 5). The characters with the greatest weight are frequency of mention of the taxa *Agaricus* spp., *Hypomyces lactifluorum* (Schwein.) Tul. & C. Tul., and *Laccaria* spp. Contrastingly, frequencies of mention of *Pleurotus djamor* (Rumph. ex Fr.) Boedijn*, Schizophyllum commune* Fr., and *Favolus tenuiculus* P. Beauv. are the characters of greatest weight for the discrimination of Pantelho and Chenalho. The second principal component explains 60.96% of the variability discriminating Zinacantan, east Chamula, and center-west Chamula from San Cristobal de Las Casas and Huixtan. The characters of greatest weight are the frequency of mention of the taxa *Turbinellus floccosus* (Schwein.) Earle ex Giachini & Castellano*, Lactarius indigo* (Schwein.) Fr., *Lactarius deliciosus* (L.) Gray, and *Daldinia* spp.

In the case of toxic mushrooms, considering all study sites, people mentioned 15 taxa (Table 5). Through photographic stimuli, 17 taxa were recognized (Table 6).

**Table 5:** Frequencies of mention of the toxic ethnotaxa registered in the study sites

Taxa	No. Men.	rel FM	rel FM. M	rel FM. W	Dif. M-W	rel FM. F	rel FM. NF	Dif. F-NF	rel FM. E	rel FM. NE	Dif. E-NE
*Agaricus* spp	2	1.50	1.61	1.41	0.20	3.51	0.00	3.51	1.28	1.82	−0.54
*Amanita arocheae* Tulloss, Ovrebo & Halling	12	9.02	6.45	11.27	−4.82	7.02	10.53	−3.51	3.85	16.36	−12.52
*Amanita bisporigera* G.F. Atk.	3	2.26	0.00	4.23	−4.23	1.75	2.63	−0.88	0.00	5.45	−5.45
*Amanita flavoconia* G.F. Atk.	5	3.76	4.84	2.82	2.02	0.00	6.58	−6.58	1.28	7.27	−5.99
*Amanita muscaria* (L.) Lam.	56	42.11	37.10	46.48	−9.38	47.37	39.47	7.89	42.31	40.00	2.31
*Amanita phalloides* (Vaill. ex Fr.) Link	1	0.75	0.00	1.41	−1.41	1.75	0.00	1.75	0.00	1.82	−1.82
*Amanita virosa* Bertill. y *A. verna* (Bull.) Lam.	10	7.52	9.68	5.63	4.04	7.02	7.89	−0.88	6.41	9.09	−2.68
*Suillellus luridus* (Schaeff.) Murrill	32	24.06	16.13	30.99	−14.86	26.32	22.37	3.95	17.95	32.73	−14.78
*Coprinopsis atramentaria* (Bull.) Redhead, Vilgalys & Moncalvo	1	0.75	0.00	1.41	−1.41	0.00	1.32	−1.32	0.00	1.82	−1.82
*Hypholoma fasciculare* (Huds.) P. Kumm.	5	3.76	4.84	2.82	2.02	5.26	2.63	2.63	3.85	3.64	0.21
*Inocybe rimosa* (Bull.) P. Kumm.	1	0.75	0.00	1.41	−1.41	1.75	0.00	1.75	1.28	0.00	1.28
*Psilocybe* spp.	3	2.26	1.61	2.82	−1.20	1.75	2.63	−0.88	0.00	5.45	−5.45
*Ramaria formosa* (Pers.) Quél.	5	3.76	1.61	5.63	−4.02	3.51	3.95	−0.44	0.00	9.09	−9.09
*Russula emetica* (Schaeff.) Pers.	19	14.29	12.90	15.49	−2.59	26.32	5.26	21.05	15.38	12.73	2.66
*Scleroderma areolatum* Ehrenb.	5	3.76	0.00	7.04	−7.04	3.51	3.95	−0.44	1.28	7.27	−5.99

*No. Men.* number of mentions, *FM rel* relative frequency of mention, *rel FM. M* relative frequency of mention men, *rel FM. W* relative frequency of mention women, *rel FM F* relative frequency of mention field-linked occupation, *rel FM NF* relative frequency of mention non-field-linked occupation, *rel FM E* relative frequency of mention formal education, *rel FM NE* relative frequency of mention without formal education, *Dif.M-W* difference between men and women, *Dif. F-NF* difference between field-linked occupation and non-field-linked occupation, *Dif.E-NE* difference between formal education and without formal education

**Table 6:** Names assigned to toxic mushrooms in the study sites. The most common names are in bold letters

Species	Central-western Chamula	Eastern Chamula	Chenalho	Huixtan	Pantelho	San Cristobal de Las Casas	Zinacantan
*Amanita virosa* Bertill. y *A. verna* (Bull.) Lam.	***Sakil yuy***	*Poxil vov, Yuy jmilvanej*	*Sakil yuy*	*Sakil yuy*	*Sakil chikin te’*	*Sakil yuy*	*Sakil yuy*
*Amanita phalloides* (Vaill. ex Fr.) Link	***Vixil yuy***	*Yuy jmilvanej*, *Poxil vov*		*Yuy ka’*		*Yat ka’*	*Yuy jmilvanej*
*Amanita bisporigera* G.F. Atk.	***Sakil yuy*** *, Yat ka’*	*Sakil yuy*		*Sakil yuy*			*Sakil yuy, Yat ka’*
*Amanita arocheae* Tulloss, Ovrebo & Halling	***Cholchol be*** *jmilvanej, Yat ka’*	*Cholchol be jmilvanej, Yat ka’, Yuy ka’*		*Yuy ka’*		*Cholchol be* de veneno	*Yat ka’, Chamel te’*
*Amanita flavoconia* G.F. Atk.	***Yuy jmilvanej***	*Yuy jmilvanej*		*Yuy* de veneno		*Yuy* venenoso	*Yuy jmilvanej*
*Galerina marginata* (Batsch) Kühner	***Chechev jmilvanej***	*Chechev jmilvanej*					San Andrés de veneno
*Coprinopsis atramentaria* (Bull.) Redhead, Vilgalys & Moncalvo		*Chichal ka’, Yat ka’*					*Yat ka’*
*Amanita muscaria* (L.) Lam.	***Yuy chauk*** *, Tsajal chechev*	*Yuy chauk*, *Poxil vov*	*Yuy chauk*	*Yuy chavuk*	*Yuy chauk*	*Yuy chauk*	*Yuy chauk, Tsajal yuy*
*Russula emetica* (Schaeff.) Pers.	*Sat chij, Yat ka’*	***Chuch chij***	*Tsajal yuy*	*Tsajal chuch*			
*Inocybe rimosa* (Bull.) P. Kumm.						*Yat ka’*	
*Hypholoma fasciculare* (Huds.) P. Kumm.	***K’anal chechev jmilvanej***	*K’anal chuch jmilvanej*	*K’anal chin te’* de veneno	*Chechev* de veneno			*Chechev* veneno
*Cortinarius orellanus* Fr.				***Osorio*** *chuch*			
*Psilocybe* spp.	*Yat ka’*	*Yat ka’*					
*Scleroderma areolatum* Ehrenb.	***Sat pukuj***	*Sat pukuj*		*Chinam tsi*		*Sat pukuj*	*Cholchol be* veneno
*Suillellus luridus* (Schaeff.) Murrill	***Sekub t’ul jmilvanej***	*Pan chuch jmilvanej*		*Ik’al p’ukuts*		*Sekub t’ul* venenoso	*Sekub t’ul jmilvanej*
*Ramaria formosa* (Pers.) Quél.	***Yisim chij jmilvanej***						
*Agaricus xanthodermus* Genev.		***Moni’ jmilvanej***					

Only two toxic ethnotaxa are under-differentiated. *Amanita virosa* Bertill. and *Amanita verna* (Bull.) Lam. are identified as the ethnotaxon *sakil yuy*, and some of the population even includes *Amanita bisporigera* G.F. Atk. under this ethnotaxon because of its mostly white color. The different species of the genus *Psilocybe* are categorized under the same ethnotaxon following the criterion of growth site, in this case, animal excrement. The other 13 taxa have a one-to-one correspondence with Linneaean species. For 37.6% of the interviewed population, toxic mushrooms have no proper identifying name as edible species do; no information was provided on this matter. Some people cited the name *yat ka’* (meaning “horse penis”) to refer in general to any toxic mushroom.

Toxic mushroom names assigned to these species in the studied Tsotsil communities are listed in Table 6.

The most frequently mentioned toxic mushroom ethnotaxa were *yuy chauk* (*Amanita muscaria* (L.) Lam.), which was mentioned 56 times, *sekub’t’ul jmilvanej* (*Suillellus luridus* (Schaeff.) Murrill), mentioned 32 times, and *chuch chij* (*Russula emetic* (Schaeff.) Pers.) men, mentioned 19 times (Table 5). *Yuy chauk* is mentioned by 42.11% of the interviewed population. *Yuy chauk* is mentioned by 42.11% of the total interviewed population. Considering all of the participants, the average number of mentioned toxic species is 1.20. Seven ethnotaxa is the highest number of mentioned species by any of the interviewed people. Only three toxic species are mentioned by more than 10% of the population.

When comparing the toxic species mentioned by men and women, the number of ethnotaxa mentioned by either group does not significantly differ (*U* = 0.1997, *P* > 0.05) (Table 3). The number of times each ethnotaxon is mentioned by men and women does not significantly differ either (*χ*^2^ = 19.398, *P* = 0.1503) (Table 3).

When comparing occupation with regard to knowledge of toxic mushrooms, the number of mentioned ethnotaxa by people working in the fields and those not working in the field is not significantly different (*U* = 0.1754, *P* > 0.05) (Table 3). However, the number of times each ethnotaxon is mentioned does differ significantly between these groups (*χ*^2^ = 30.385, *P* = 0.0068) (Table 3). For example, *Russula emetica* is mentioned 21% more frequently by people working in the field (Table 5).

When we consider the schooling of the interviewed population, the number of mentioned ethnotaxa does not significantly differ between people with and without formal education (*U* = 0.0627, *P* > 0.05) (Table 3). However, significant differences appear between these groups when comparing how many times each ethnotaxon is mentioned (*χ*^2^ = 32.664, *P* = 0.0032) (Table 3). *Suillellus luridus*, for example, is mentioned 14% more frequently by those without schooling than by those with.

**Fig. 6:**
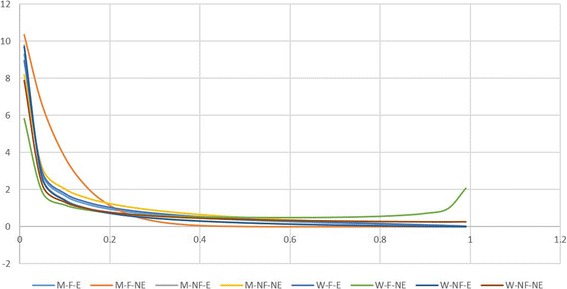
Probability density of the relative number of known toxic mushrooms. Model including different sociodemographic features. M-F-E = Men-occupation linked to Field- with Education, M-F-NE = Men-occupation linked to Field-without Education, M-NF-E = Men-occupation Not linked to the Field-with Education, M-NF-NE = Men-occupation Not linked to the Field-without Education, W-F-E = Women-occupation linked to Field- with Education, W-F-NE = Women-occupation linked to Field-without Education, W-NF-E = Women-occupation Not linked to the Field-with Education, W-NF-NE = Women-occupation Not linked to the Field-without Education

Looking at different models to explore the interaction of sociodemographic conditions for toxic mushrooms, models considering the interaction between schooling and either gender or occupation are better supported (Table 4). Thus, there is a higher probability of finding unschooled field workers who know a higher number of toxic species (Fig. 6).

**Fig. 7:**
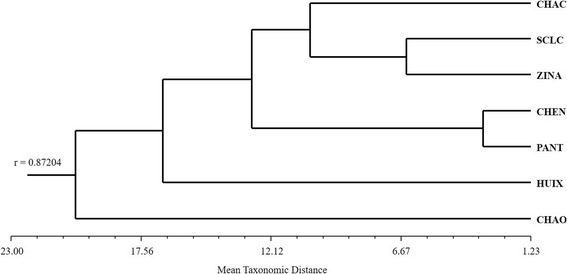
Cluster analysis of the four study sites using the Average Taxonomic Distance index based on the relative frequency of mention of toxic mushrooms

**Fig. 8:**
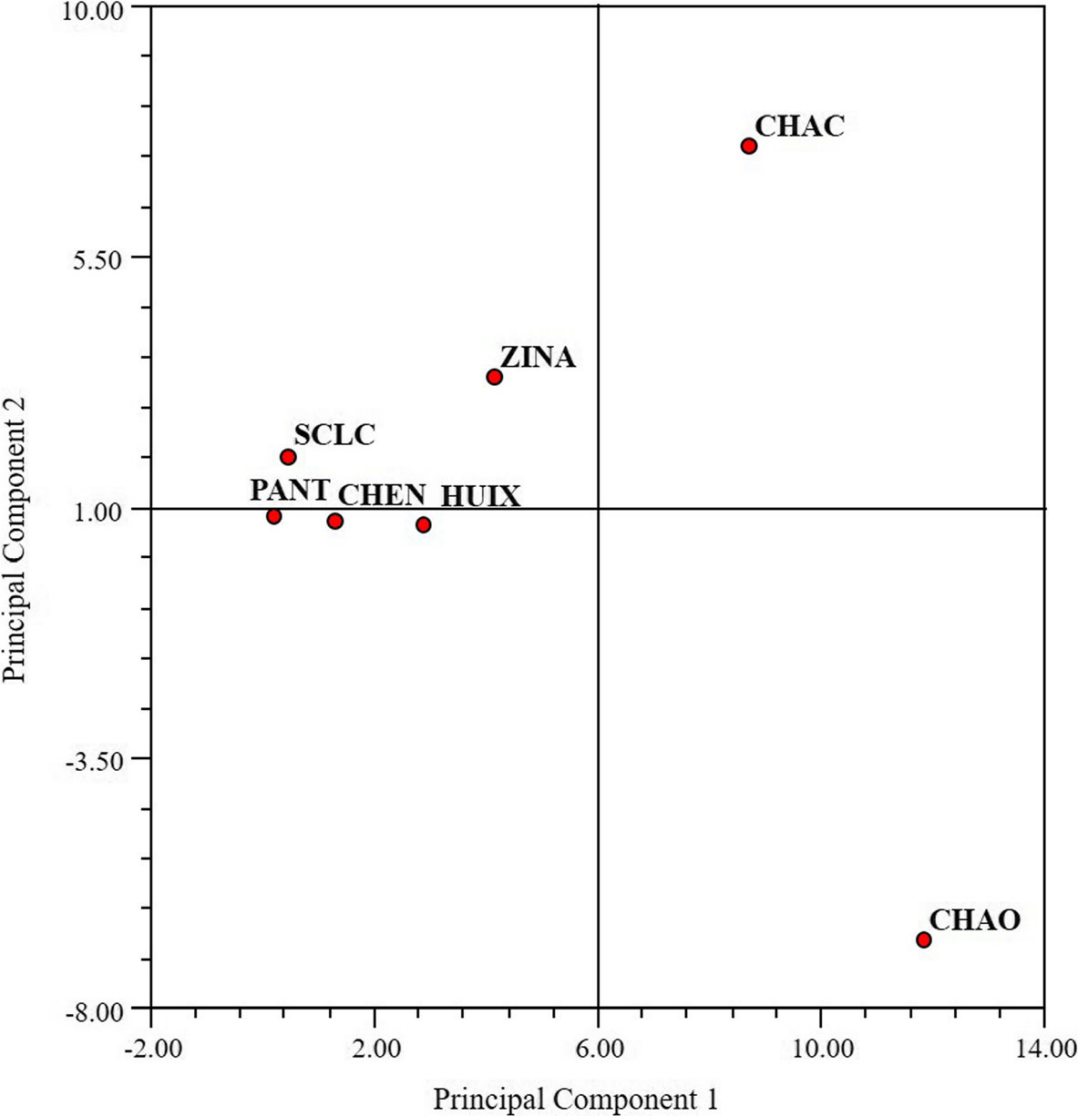
Principal component analysis for the studied communities based on the relative frequency of mention of toxic mushrooms

The classification analysis was based on the relative frequency of mention of toxic mushroom groups on the one hand center-west Chamula, San Cristobal de Las Casas, and Zinacantan and on the other hand Chenalho, Pantelho, and Huixtan and east Chamula as the most different communities (Fig. 7). The PCA shows that the first principal component explains 45.48% of the variation, discriminating the Chamula communities from the group that includes San Cristobal de Las Casas, Pantelho, Chenalho, Huixtan, and Zinacantan (Fig. 8). The characters with greatest weight are the frequencies of mention of *Amanita bisporigera*, *Scleroderma areolatum* Ehrenb., and *Psilocybe* spp. The second principal component, explaining 66.81% of the variation, discriminates east Chamula from all other sites. The characters of greatest weight are the frequencies of mention of *Russula emetica*, *Amanita phalloides* (Vaill. ex Fr.) Link, and *Amanita virosa* and *A. verna*.

## Discussion

The results show part of the Tsotsil mycological knowledge of the named and recognized species of edible and toxic species. In some areas of Latin America, and particularly in Mexico, the distribution and cultural significance of edible mushrooms has been explored in depth [8], which makes it possible to find patterns or contrasting situations in the usage of this resource. However, toxic mushrooms have been intermittently studied at best, and in consequence, there is scarce information on this subject, both in mycology (knowledge of their diversity and distribution) and in ethnomycology.

Regarding the number of mentioned taxa in the free listing exercises, the 25 edible ethnotaxa are an indicator of the relevance of these organisms as a dietary product among Tsotsil communities in the Highlands of Chiapas. This number surpasses the 18 ethnotaxa reported by Shepard et al. [41] among Tsotsil groups. Furthermore, contrasting the degree of significance of edible mushrooms in this region with that of other areas in the country, it can be appreciated that they are surpassed only by the 28 ethnotaxa reported in the Sierra Nevada region in Estado de Mexico, central Mexico [54]. They are above the 21 ethnotaxa registered in Ixtlan de Juarez, Oaxaca in southern Mexico, the 16 reported in two municipalities of the Tarahumara mountain range in Chihuahua (northern Mexico), or the 13 reported in a Michoacan community in western Mexico (all of these regions have similar vegetation types and mycobiotas in general terms) [14, 55, 56]. When we compare this number with those obtained studying other Mayan peoples from temperate zones, it surpasses the ethnotaxa mentioned by Tojolabals (14), Chuj (12), and Tseltals (17) [35, 41, 42]. While these comparisons are a parameter for the degree of knowledge of mycological diversity in the studied groups, it should be pointed out that free listings do not account for the total ethnotaxa known by people, but rather those that are most significant [14]. Furthermore, it is highly probable that not all the species within an ethnotaxon have been identified. Consequently, the number of edible species might still grow as further ethnomycological research is carried out. Be that as it may, the 25 edible ethnotaxa that are registered in this study accounts for around 7% of the 371 edible taxa reported for Mexico [8]; this points to a rich mycocultural heritage in this region.

It is also remarkable that species such as *Morchella* sp. or *Ustilago maydis* (DC.) Corda are not named in free listings. Although these species are of great significance in other regions of the country and the world, reaching quite high prices in markets, among the Tsotsil, they are not traditionally used [40].

Regarding toxic species, not many studies have documented elements from this category through free listings. The 15 ethnotaxa mentioned in this study surpass the 14 recorded by a mestizo community and the 11 mentioned by a Nahua community both in the state of Tlaxcala in sites with a similar mycobiota [18]. In the highlands of Chiapas, there is previous work by Shepard et al. [41], which recorded three mentioned toxic ethnotaxa, in addition to the residual term *yat ka’*.

Along with the number of recognized taxa, the degree of significance of mushrooms can be documented by studying taxonomy and classification systems and applying linguistical analysis. According to Turner [57], the most culturally significant organisms will have simple, non-analyzable names. In Tsotsil systematics of edible mushrooms, the three species with these features are also the most frequently mentioned using free listing techniques: *yuy*, *moni’*, and *chechev*.

Furthermore, 48% of the mentioned ethnotaxa have a one-to-one correspondence as is defined by Berlin et al. [53]; that is to say, a single local generic taxon corresponds to a single Linnean species. Another 48% of ethnotaxa are subdifferentiated, that is, a local name corresponds to different species. For example, the ethnotaxon *yisim chij* is valid for different species, such as *Ramaria* cf. *cystidiophora*, *Ramaria* subgen. *Laeticolora*, and other not yet identified species [45]. The ethnotaxon *Yok wakax* is valid for both *Hydnum rufescens* Pers. and *Hydnum repandum* L. Meanwhile, the ethnotaxon *Sekub t’ul* corresponds to species of the genus *Boletus* like *Boletus pinophilus* Pilát & Dermek and *Boletus atkinsonii* Peck. as well as species from the genus *Suillus* such as *Suillus tomentosus* Singer and *Suillus placidus* (Bonord.) Singer. In these cases, identified species within these ethnotaxa are a product of study cases in the Tsotsil area [41, 45] and the author’s own experience. The use of a photograph catalog for taxonomic corroboration of the ethnotaxa is a handy methodological strategy [45, 58], but it does not exactly determine the taxonomic identity of the species within any given ethnotaxon. However, it is likely that further ethnomycological works with more extensive collections new species will be found within these ethnotaxa. The case of the ethnotaxon *yuy* is of particular interest; among the interviewed population in Chamula, some recognize two types of this species: *yuy* and *k’antsu* or *tsajal yuy*, which have a one-to-one correspondence with *Amanita hayalyuy* and *Amanita jacksonii* respectively. However, for most of the interviewed in the rest of the municipalities, both species are known simply as *yuy*. *Armillaria mellea*, on the other hand, is conceptualized as different ethnotaxa based on substrate, phenology, and even size; however, most people do not pay attention to such details and name it as a single species. While this study was not designed to evaluate loss of knowledge, we observed only a small portion of the population with such a precise taxonomical knowledge.

Along with the non-analyzable names that are mentioned above, other names for the ethnotaxa are assigned for their morphological resemblance to elements from everyday life among the Tsotsil or other criteria, such as animal parts (*sekub t’ul* or rabbit’s liver; *yok wakax* or cow’s tongue, *yisim chij* or goat’s beard), a species’ characteristic colors (*yaxal manayok* or blue manayok, *k’anal manayok* or yellow *manayok*), vegetable species serving as substrate (*checheval mail* or gourd—*Curcubita* sp.—mushroom, *checheval tulan* or oak—*Quercus*—mushroom). According to Berlin et al. [53] in ethnobiological nomenclature morphological, anatomical or ecological features are generally used by associating them to biological referents. Some of these names had been previously reported by Shepard et al. [41] in the Tsotsil region.

It is remarkable to find the name *sakitaj* for *Pleurotus djamor* since it is a term present in many Mayan languages both in Chiapas (Tsotsil, Tseltal, Mam, Tojolab’al, and Chuj) [35, 37–39, 41–43, 59, 60] and in Guatemala [61, 62]. Without going much further into this subject, the fact that this term is the same in Tselatal, Tsotsil, Tojolab’al, and Chuj, as well as all the Mayan languages from the Western branch (Western Maya, WM) originated hypothetically about 30 centuries ago [63], may indicate that these species was named and used at least ever since 1000 BC. Furthermore, Kaufman [63] points out that the term *K’an tsu*, which refers to the species *Amanita jacksonii*, originates in reconstructions like those of the Central Maya language (CM), which existed some 36 centuries ago, around 1600 BC. This backs the hypothesis that these mushrooms and their use as food are quite ancient.

As for toxic mushrooms, the observed patterns are different. According to Hunn [10], only a part of the natural discontinuities is recognized in ethnobiological classifications and consequently all non-classified entities are recognized in general terms. To other authors, only those organisms closer to human life or those arising some kind of interest will be included in their systematic [21, 64, 65]. Among the interviewed Tsotsil, almost 4 out of 10 explicitly say that toxic mushrooms have no name or only refer to them with a general term that could be cataloged as a “residual category” as was proposed by Hunn [10]. This pattern had been previously reported among Chuj and Tojolab’al people in Chiapas [42]. In those groups, more than half of the interviewed people lacked names for toxic mushrooms. This indicates toxic mushrooms do not seem to be of practical interest in the life of Tsotsil and, apparently, other Mayan groups. This does not mean that to them toxic species are unimportant per se (they can certainly cause severe damage to health or even death if they are consumed), but rather people are unconcerned to learn the specific features of toxic species and recognize them as particular ethnotaxa. Instead, they recognize them by differentiating them from edible species. In most cases, when toxic ethnotaxa were named an obligated reference is made to the edible species that is similar to them. In some cases, the adjective *jmilvanej* which means “murderer” is added to indicate the toxic nature of some species, such as: *chechev jmilvanej* (*Galerina marginata* (Batsch) Kühner), *sekub t’ul jmilvanej* (*Suillelus luridus*) or *yisim chij jmilvanej* (*Ramaria formosa* (Pers.) Quél.). These ethnotaxa have names that make reference to their edible counterpart: *chechev* (*Armillaria mellea*), *sekub t’ul* (*Boletus* spp. y *Suillus* spp.), and *yisim chij* (*Ramaria* spp.) respectively. In other cases, adjectives make reference to morphological features that set them apart from edible species. Some examples are the ethnotaxa *sakil yuy* (*Amanita virosa* and *A. verna*) which point out the white color they have as opposed to the edible *yuy* (*Amanita hayalyuy* and *A. jacksonii*), or *yuy chauk* (*Amanita muscaria*), which point to differences in its origin. This mushroom is said to be a type of *yuy* that is originated by thunderbolts. Few toxic ethnotaxa with a name make no reference to edible species, but rather focus on specific features of the toxic species, this is the case of the ethnotaxno *chuch chij*, which can be translated as sheep mushroom (*Russula emetica*). It is so named because it is found and eaten by sheep taken out to graze by Tsotsil women [45]. Furthermore, *Amanita arocheae* is called *cholchol be*, which means “grooves in the road”, making reference to spaces where its fruiting bodies can be found. It is important to point out that there are no simple, non-analyzable names for toxic mushrooms, which, as was pointed out earlier, can be considered an indicator of cultural significance for the taxa that do. Ramirez-Terrazo [18] found this same pattern of toxic mushroom local knowledge being built in comparison to edible mushroom knowledge when working with Nahua and mestizo people in the central Mexican state, Tlaxcala.

*Suillelus luridus*, *Russula emetica*, and *Amanita muscaria* are regionally reported to be toxic species. However, based on findings from other studies, some considerations to this condition should be mentioned. *Suillelus luridus* is consumed as food in Poland and other east Europe countries, as well as in Estado de Mexico [66–68]. *Russula emetica* is also eaten in some regions in India and Russia and in east Europe countries such as Bulgaria [2, 69]. Even *Amanita muscaria* has been reported to be consumed as food in east Europe countries and some regions in Russia and Japan [70]. In all of these instances, the authors mention that these mushrooms should be eaten only after parboiling them with vinegar and discarding the water. This is due to the fact that the toxins responsible for gastrointestinal intoxications in the first two species and the ibotenic acid in *Amanita muscaria* are water soluble [18, 71]. Nonetheless, it is paramount to be certain of the taxonomic identity of the cited species as well as the chemical features of their secondary metabolites, both in sites where they are reported to be eaten and in places where they are deemed toxic, so that we may conclusively affirm that they are the same species [67].

Frequency of mention has been a widely used indicator to recognize species with the greatest cultural significance, not only in the case of mushrooms, but also to analyze other organisms [14–17]. In the case of edible mushrooms, even though species reported in other regions of Mexico vary in composition according to free listings, some of the most frequently mentioned taxa in the Highlands of Chiapas coincide with those of different zones of the country. A clear example is set by *Amanita caesarea* s.l., *Agaricus* spp., and *Ramaria* spp. These are the most frequently mentioned species in this study, and they are also among the most mentioned in studied sites in central, south, and west Mexico [14–16, 56, 72]. These groups of species appear to be some of the most important not only in this country, but in the world, since they are reported to be eaten in many countries [2]. *Armillaria mellea* is a different matter; even though it is the third most mentioned species in this study, it does not appear in listings carried out in other regions of Mexico. The case of *yuy* in the Highlands of Chiapas is remarkable, since it is not only the most mentioned taxon, but also one of the most consumed and commercialized species in regional markets [44]. Garibay-Orijel and Ruan-Soto [8] point out that *Amanita caesarea s.l.* is the most important edible species, its consumption is reported in more than 50 ethnomycological studies throughout the country. In contrast, species that are commonly mentioned in other regions like *Lyophyllum decastes* (Fr.) Singer, *Russula brevipes* Peck and *Morchella esculenta* (L.) Pers. [15, 16, 56, 72] appear not to be consumed, or at least not importantly so in the studied region.

It would seem, according to evidence in different ethnomycological studies carried out in similar vegetation zones, that knowledge of edible species is not homogeneous in any given population and that only a few species are recognized by over 50% of the people. In this study, only five ethnotaxa are mentioned by over 50% of the population, while the reported number is four in central and south Mexico [14–16] and five in the west of the country [56]. People mention an average of 6.24 species in free listing exercises; this is a relatively high count for this state [44], but it still is below average when compared to central Mexico, where as many as 15 species are the average [15].

The composition of mentioned toxic species, on the other hand, differs from that reported by Ramirez-Terrazo [18] in Nahua and mestizo communities in Tlaxcala, in central Mexico. This may be due to the particular perception each people have about the toxic nature of uneaten species. However, we must not disregard the scarce knowledge we currently have of toxic mycobiota in Mexico [20]. Both in this study and that of Ramirez-Terrazo [18], *Amanita muscaria* is the most mentioned toxic species. The case of *Suillelus luridus*, the second most frequently mentioned species, is also noteworthy. Its relevance may be due to the fact that during the year in which this study as carried out, at least three cases of intoxications related to consumption of this species occurred in the region and they were shared in communication media. To Albuquerque (pers.com.) in the process of recuperating and keeping biocultural information, free listings record information from events of a frequent and contemporary nature. While no solid evidence has been published to support this claim, it is plausible that the same phenomenon occurs with toxic mushrooms. In this significance category, knowledge is centered in three species mentioned by more than 10% of the interviewed subjects; no species is mentioned by more than 50% of the population. This situation is contrasting with reports from central Mexico; it appears that in that area there is a wider knowledge: 14 toxic species are mentioned, ten of them are recognized by more than 10% of the population and at least two ethnotaxa are recognized by more than 50% of the population [18].

Some authors have suggested that both the number of known species and the degree of cultural significance of species is not homogeneous within a community but rather varies across sociodemographic variables such as gender, occupation, and schooling [14, 15, 49].

In this study, both occupation and schooling seem to be related to the number of mentioned edible mushroom species. People with occupations linked to the field and people without formal education mention a significantly higher number of species than those who do not work in fields and have formal education. Peasant people share a lifestyle in direct contact with elements from nature in which resources from the wilderness are commonly used [1, 49]. On the other hand, a problem in Chiapas, and more widely in México, is that basic education instructs students in topics that are often unrelated to the reality of their communities, particularly in things related to their biocultural heritage [73]. Thus, schooling comes at the cost of failing to acquire traditional ecological knowledge. While economic and education development systems have somewhat increased material wealth in communities, they have also generated loss of traditional ecological knowledge. Studies like that of Saynes-Vasquez et al. [49] show that when cultural change, indicated by occupational activity, occurs a higher education level is associated with greater loss of ethnobotanical knowledge. Now, regarding gender, while many ethnomycological studies cite the transcendental role of women in wild mushroom use [74–76], this study found no significant differences in number of mentioned species by gender. The amount of times that each ethnotaxon is mentioned by the interviewed population does present significant differences when comparing gender, occupation, and schooling. Women mention *Armillaria mellea* significantly more than men. This may be related to the fact that this species appears in the base of *Quercus* trees; these species are used for firewood among the Tsotsil and it is generally women who collect this resource for everyday use [77].

Contrastingly, no significant differences were detected in any of the sociodemographic variables regarding the number of toxic species that were mentioned. Again, the number of times each ethnotaxon is made reference to is different. For example, *Russula emetica* is mentioned significantly more by those dedicated to field-related activities. Among the Tsotsil, sheep herding is a central activity [78]. When peasant people take their sheep grazing, they frequently observe the animals consume *Russula emetica*, which gives this mushroom its local name: *chuch chij* or sheep mushroom. People employed in secondary or service-related economic activities do not possess this knowledge.

When we compare edible species knowledge between the different studied localities through a PCA, Pantelho and Chenalho form a separate group from all other Tsotsil communities because they mention species like *Schizophyllum commune*, *Pleurotus djamor*, and *Favolus tenuiculus* more often. Because of their altitude, around 1500 m a.s.l., the knowledge pattern in these municipalities is more akin to those in tropical communities, where saprobial species are preferred for consumption [44, 79]. On the other hand, in other Tsotsil localities, mycorhizogenous species, such as *Laccaria* spp. and *Hypomyces lactifluorum*, are mostly mentioned, which falls into the same pattern reported for other temperate areas in Chiapas and all of Mexico [14, 15, 35, 42].

When the PCA was performed to analyze toxic mushroom knowledge, both Chamula localities are separated from all others because species like *Amanita bisporigera* are more frequently mentioned in them. In recent years, this species has been present in different communication media as the cause for deadly intoxications, especially in Chamula municipality, which has naturally led to it being one of the most mentioned species in this zone.

## Conclusions

According to currently available evidence, the Tsotsil region of the Highlands of Chiapas appears to be the region in this state where the most mushroom species are recognized on average. When compared nationwide, people in this region recognizes 14% more species than do people from southern Mexico, 32% more than do those from northern Mexico, 42% more than do those from western Mexico, and 53% more than do those from Eastern Mexico. They are only 11% below knowledge from communities in temperate regions in central Mexico who have the greatest reported mushroom knowledge in the country.

Furthermore, when considering the initial hypothesis, we can appreciate schooling and occupation are the most determinant conditions for people to recognize more or less mushroom species, while gender is not. People with a lower formal education and occupations linked to the field are bound to mention more edible species. In the rural context of the Highlands of Chiapas, and perhaps the whole country, the formal education system does not have synergy with traditional mycological knowledge. Thus, attendance to formal school settings may be equivalent to renouncing knowledge of these species. Moreover, occupations diverting rural population to other productive activities, while bringing greater material wealth, seem to be in detriment of traditional mycological knowledge as well. However, it is noteworthy that certain specific species are more often mentioned by some of these social subsets, as is the case of *Armillaria mellea*, more frequently mentioned by women in general.

The identifying criteria setting edible species apart from their toxic counterparts seem not to be established based on observation of particular features of toxic species or warnings in their local names, as is the case in academic mycology, but rather in a precise and profound knowledge of the features of edible species. Thus, the strategy to avoid intoxication is not recognizing species as toxic, but instead “ignoring” these species in general or naming them by comparisons with their edible simile. Attention is focused on edible species, their naming, feature identification, and, evidently, their collection. The process of distinguishing a toxic species from one that is edible is of utmost importance and it should doubtlessly continue to be studied in greater depth in this region of Mexico.

According to the evidence we gathered, the ethnotaxa with the highest cultural significance are *yuy* (*Amanita hayalyuy* and *A. jacksonii*), *moni’* (*Agaricus* spp.), *chechev* (*Armillaria mellea*), *sakitaj* (*Pleurotus djamor*), *yisim chij* (*Ramaria* spp.), and *sekub t’ul* (*Boletus* spp. and *Suillus* spp.). It is probable that people seek these species when they forage for wild mushrooms and that toxic species resembling these taxa are the most potentially dangerous for collectors. Species like *Amanita bisporigera*, *Agaricus xanthodermus*, *Galerina marginata*, and *Hypholoma fasciculare*, *Ramaria formosa*, or *Suillelus luridus* may be the toxic species that future research and prevention programs should focus on. Meanwhile, species like *Amanita muscaria* are widely recognized by the population. The reasons behind this apparent contradiction to the observed pattern are an interesting aspect to focus further study on. It seems to relate to worldview aspects of this human groups and the relationship that it has with lightning, a profoundly significant element of this people’s culture.

This information about toxic species is of great usefulness to focus future prevention strategies on providing the collectors with the basic features of these species. Furthermore, it could lead to more effective pre-attention strategies when taking action in communities where intoxications have happened and even in hospital attention, by focusing in the specific syndromes caused by each group of toxic mushrooms and the proper therapeutic measures a medic should apply.

There are many research lines in ethnomycology that need attention in this region; however, studies like the one that is presented here can contribute to better planning in public health to prevent and avoid wild mushroom poisonings as much as possible.

## Abbreviations

m a.s.l.: Meters above sea level
NTSYS: Numerical Taxonomy and Multivariate Analysis System
PCA: Principal component analysis
UPGMA: Unweighted Pair Group Method with Arithmetic Mean
